# Use of previous-day recalls of physical activity and sedentary behavior in epidemiologic studies: results from four instruments

**DOI:** 10.1186/s12889-019-6763-8

**Published:** 2019-06-03

**Authors:** Charles E. Matthews, David Berrigan, Beate Fischer, Sjaan R. Gomersall, Andrea Hillreiner, Youngwon Kim, Michael F. Leitzmann, Pedro Saint-Maurice, Timothy S. Olds, Gregory J. Welk

**Affiliations:** 1 Division of Cancer Epidemiology and Genetics, National Cancer Institute, Bethesda, MD USA; 2 Division of Cancer Control and Population Sciences, National Cancer Institute, Bethesda, MD USA; 3 Department of Epidemiology and Preventive Medicine, University of Regensburg, Regensburg, DE Germany; 4 School of Health and Rehabilitation Sciences, The University of Queensland, Brisbane, AU Australia; 5 Department of Health Kinesiology and Recreation, University of Utah, Salt Lake City, UT USA; 6 MRC Epidemiology Unit, University of Cambridge School of Clinical Medicine, Cambridge, UK; 7 School of Health Sciences, University of South Australia, Adelaide, AU Australia; 8 Department of Kinesiology, Iowa State University, Ames, IA USA

**Keywords:** Energy expenditure, Sitting time, Behaviour change, Measurement, Exposure assessment, Ecological momentary assessment, Public health

## Abstract

**Background:**

The last few years have seen renewed interest in use-of-time recalls in epidemiological studies, driven by a focus on the 24-h day [including sleep, sitting, and light physical activity (LPA)] rather than just moderate-vigorous physical activity (MVPA). This paper describes four different computerised use-of-time instruments (ACT24, PAR, MARCA and cpar24) and presents population time-use data from a collective sample of 8286 adults from different population studies conducted in Australia/New Zealand, Germany and the United States.

**Methods:**

The instruments were developed independently but showed a number of similarities: they were self-administered through the web or used computer-assisted telephone interviews; all captured energy expenditure using variants of the Ainsworth Compendium; each had been validated against criterion measures; and they used a domain structure whereby activities were aggregated under categories such as Personal Care and Work.

**Results:**

Estimates of physical activity level (average daily rate of energy expenditure in METs) ranged from 1.53 to 1.78 in the four studies, strikingly similar to population estimates derived from doubly labelled water. There was broad agreement in the amount of time spent in sleep (7.2–8.6 h), MVPA (1.6–3.1 h), personal care (1.6–2.4 h), and transportation (1.1–1.8 h). There were consistent sex differences, with women spending 28–81% more time on chores, 8–40% more time in LPA, and 3–39% less time in MVPA than men.

**Conclusions:**

**Although there were many similarities between instruments, d**ifferences in operationalizing definitions of sedentary behaviour and LPA resulted in substantive differences in the amounts of time reported in sedentary and physically active behaviours. Future research should focus on deriving a core set of basic activities and associated energy expenditure estimates, an agreed classificatory hierarchy for the major behavioural and activity domains, and systems to capture relevant social and environmental contexts.

## Background

Systematic methods to characterize how people use their time were developed as early as the 1920’s [1] and this general approach was coupled with the first field-based measures of oxygen consumption in the 1940s to estimate daily energy requirements, and the endurance limits of industrial workers [2]. Thus, our earliest methods to estimate free-living physical activity and energy expenditure relied heavily on the assessment of time spent in a variety of behaviors, and it was noted that to estimate energy expenditure “…larger errors are likely to arise from a failure to determine correctly the length of time spent in any activity rather than in any assessment of the metabolic cost of that activity” [2], highlighting the critical importance of time in epidemiological and behavioral research on physical activity.

These early field-based assessments of energy expenditure formed the foundation for future efforts to standardize coding of physical activity and exercise behaviors. It also established the basis upon which self-reported physical activity measurement methods evolved, including refinements of the diary-based approach [3, 4], the development of seven-day recall interviews [5, 6], and ultimately questionnaires designed to assess habitual physical activity levels for research and surveillance (e.g., [7–10]). While questionnaires have been invaluable in developing an epidemiological evidence base for the health benefits of leisure-time physical activity/exercise [11, 12] and health risks associated with selected sedentary behaviors (e.g., television) [13, 14], questionnaires are often limited by reporting errors [15–17] and they rarely capture the full spectrum of sedentary behavior and light intensity physical activities, the two categories of behavior in which adults spend most of their time [18, 19]. Taking cues from nutritional epidemiologists [20] and time-use researchers [21] regarding the value of the previous-day recall estimates of behavior, physical activity researchers have recently returned to a time-use oriented approach for a number of reasons: 1) As a way to fill gaps in knowledge regarding links between physical activity, sedentary behavior and health that are not adequately assessed using questionnaire-based methods [22, 23], 2) To enhance understanding of how use of time changes in response to exercise participation [24, 25], 3) To better understand the contextual determinants of physical activity and time use [26–28], and 4) In response to a shift towards a 24-h day paradigm of understanding the relationship between time use and health [29].

In this report, we describe four validated previous-day recall instruments that are being used in large epidemiologic studies and their respective approaches to estimate physical activity, sedentary behavior, and energy expenditure in adults. Our primary goal is to illustrate the methods and summary results for selected previous day recall instruments rather than to make explicit quantitative comparisons between the populations under study. To help identify areas for possible methodologic improvements in the future we also describe the similarities and differences between instruments and their scoring methods.

## Methods

We first describe the individual previous-day recall instruments and the study populations evaluated in this report (see also Table 1), followed by a general description of the time use estimates derived from each instrument.

**Table 1.** Description of previous-day recall instruments and study designs

	ACT24: PA	PAR: IA	MARCA: AU	cpar24: DE
Instrument Characteristics				
Mode of administration	self-administered via computer	computer-assisted telephone interview	computer-assisted telephone interview	self-administered via computer
Number of activities	213	270	520	262
Contextual and behavioral domains	Sleep/napping; Personal care; Household chores; Transportation; Communicating; Leisure/social; Shopping/errands/appointments; Caring/play; Work for pay; Exercise/sports/recreation; Lawn and garden; Maintenance/repair; Church/spiritual; Miscellaneous	Location: Work/Volunteer; Home Indoors; Home Outdoors; Transportation; Community Primary purpose: Work (paid job); Home/family; Volunteering; Exercise/Sports; Education; Leisure	Physical activity; Screen time; Chores; Work/study; Sociocultural; Self-care; Transport; Sleep; Quiet time	Sleep/reclining; Personal care; Food preparation/eating; Walking/transportation; Household chores; Occupational activity; Shopping/errands/appointments; Leisure/hobbies; Sports; Family and social; Outdoor; Lawn and garden; Miscellaneous
Length of recall completion	median 30 minutes	mean 20 minutes	mean 15 minutes	mean 30 minutes
Number activities reported/day	24.2 (SD=7.9)	25.3 (SD=7.6)	32.8 (SD=11.7)	23.1 (SD=9.7)
Compendium used	Ainsworth 2000	Ainsworth 2011	Ainsworth 2011; Ridley 2008	Ainsworth 2011
Assessment of Posture^a^	Yes	Yes	Yes	Yes
Sedentary classification	Sitting or reclining activities during waking day, and low energy expenditure (typically < 1.8 METs)^a^	Any waking activity ≤ 1.5 METs, regardless of body position	Any activity expected to elicit ≤ 1.5 METs undertaken in a seated or lying position while awake	Any out of bed activity ≤ 1.5 METs, regardless of body position
Light intensity activity	Non-sedentary, < 3 METs	1.6 to 2.9 METs	1.6 to 2.9 METs	1.6 to 2.9 METs
Moderate-vigorous activity	≥ 3 METs	≥ 3 METs	≥ 3 METs	≥ 3 METs
Study Design(s)				
Sample-size (N)	1,020	1,468	2,307	3,491
Age range	50-74 yrs	20-69 yrs	16-93 yrs	20-69 yrs
Location	Pittsburgh, PA USA	Iowa, USA	Australia, New Zealand	Germany
Study type	Observational	Observational	Observation; interventions	Observational
Sampling frame	Convenience sample	Stratified random sample	Convenience sample	Stratified random sample
Time-frame (calendar years)	2012-13	2014-18	2008-16	2014-present

^a^See text for details

**Activities Completed Over Time in 24 Hours (ACT24)** is an internet-based previous-day recall that was adapted from interviewer-administered recalls [30–32]. ACT24 was designed to be self-administered via the web and to estimate total time (hrs/d) spent sleeping (in bed), sedentary (sitting or reclining) and engaged in physical activity, and the energy expenditure associated with these behaviors (metabolic equivalent hours per day, MET-hrs/d) [33]. To complete ACT24, respondents add individual activities to a timeline that is segmented into four six-hour segments (midnight to midnight). They select activities from 13 broad activity categories containing a total of 213 individual activities. After an activity is selected, respondents provide additional details about each activity including the duration or start-stop time for the activity (± 5 min) and body posture while engaged in the activity (i.e., sitting, standing, some of both). Each activity reported can be translated into a variety of summary metrics including, behavioral (e.g., hrs/d sleeping, sedentary or active), in various domains of time use (e.g., hrs/d in leisure, work, transportation), and using the Compendium [34], energy expenditure (MET-hrs/d). Sedentary behaviors were defined as those performed during the waking day (out of bed) while sitting or reclining and that require little energy expenditure, typically < 1.8 METs. Consistent with domain-specific sedentary behavior assessments [33, 35, 36], and the types of sedentary behavior described on the Sedentary Behavior Research Network website [37], motorized transportation (e.g., driving or riding in a car) was classified as sedentary, even though MET values for driving are ≥2.0 METs [34, 38]. Active behaviors were those involving an upright posture, or that had higher MET levels. Data were derived from The Interactive Diet and Activity Tracking in AARP (iDATA) study [33]: a convenience sample of ambulatory adults (50–74 years) from Pittsburgh, PA who had internet access, a body mass index (BMI) < 40 kg/m^2^, and were free of major medical problems. Participants were asked to complete six ACT24 recalls over 12-months (one every other month) on randomly selected days. Signed informed consent was obtained and the study was approved by the NCI Special Studies Institutional Review Board. ACT24 was evaluated for validity using doubly labeled water and the activPAL device [33].

**The Physical Activity Recall (PAR)** survey was designed for implementation in the Physical Activity Measurement Survey (PAMS), a study designed to explore sources of measurement error in standardized PAR instruments [39, 40]. The PAR survey developed and refined for PAMS was conceptually like an established 24-h recall instrument developed by Matthews and colleagues [31]; however, adaptations were made to enable deployment through a computer-assisted telephone interviewing system. A segmented day approach was used to facilitate recall, with the previous day divided into four six-hour segments (midnight to midnight). Trained interviewers prompted participants to recall each activity they engaged in for 5 min or more on the previous day. During the recall, interviewers selected the named activity from a list of 270 activities, derived from the Compendium of Physical Activities [34], and then recorded the reported duration. Body position for a given activity was classified by the interviewer at the time of activity selection. For each activity reported, the interviewer asked for the primary Location (5 codes: Work/Volunteer, Home Indoors, Home Outdoors, Transportation, and Community) and a primary purpose (6 codes: Work (paid job), Home & Family Care, Volunteering, Exercise/Sports, Education, and Leisure) to capture context. The interviewer ensured that participants reported 360 min of activity into each of the 4 blocks to confirm complete records, but the reporting was not necessarily sequential.

Data were collected using a stratified random sample design to estimate population averages for the state of Iowa with regard to region, urbanicity and ethnicity [40]. Over 24 months, 1501 adults (21–70 yrs) who could walk, complete telephone interviews, and provide written surveys in either English or Spanish, were enrolled. Participants completed two previous day recalls about 2–3 weeks apart on randomly selected days. The length of the recalls ranged from 12 to 45 min with an average of about 20 min. Study protocols for the PAMS project were approved by the Iowa State University institutional review board. Each participant provided written informed consent before participation. The PAR was evaluated for validity in comparison to the SenseWear Armband device [40].

**The Multimedia Activity Recall for Children and Adults (MARCA)** is a computer-administered, 24-h self-reported recall tool [41, 42]. The MARCA asks participants to recall their previous day from midnight to midnight using meal times as anchor points in a segmented day format. Participants are asked to report activities in the order that they were performed in time slices of five minutes or more, by choosing from a custom compendium of over 520 activities. Each activity in the MARCA compendium, identified by a unique 6-digit activity code, captures the following data: a domain of time use, a MET value, and posture. Body posture was identified by interviewers by selecting default activities (e.g. “archery” assumes standing), or by selecting separate posture-specific versions of the same activity (e.g. “watching television – sitting” and “watching television – lying”). The time-use domains consist of nine mutually exclusive and exhaustive activity sets or “superdomains”: Physical Activity, Screen Time, Chores, Work and Study, Sociocultural, Self-Care, Transport, Sleep, Quiet Time [24]. These domains were developed by hierarchically collapsing the 520 activities in the MARCA Compendium while preserving similarity between activities and comparability with similar work. The MET values were based largely on the Ainsworth Compendium of energy expenditures for adults [34, 38] or the Ridley Compendium of energy expenditures for youth [43].

Data for this paper were drawn from 17 studies conducted in Australia and New Zealand between 2008 and 2017 using a variety of populations and a variety of sampling frames. For intervention studies, only baseline recalls were used. In most studies, the MARCA was administered by computer-assisted telephone interview (CATI) using trained interviewers, where participants were asked to recall their previous day (24-h recall) or up to two previous days (48-h recall). Participants were included in this study if they had at least one recall day available, were aged over 15 years and if data were collected using the adult version of the MARCA. All studies were approved by the relevant Human Research Ethics Committees. The MARCA was evaluated for validity in comparison to doubly labelled water [44] and the ActiGraph device [42].

**The Computer-based 24-h Physical Activity Recall (cpar24)** was developed to collect detailed information about the types, frequencies, durations, and contexts of physical activities and sedentary behaviors. The tool was designed such that it is easy to navigate and can be completed at home via the internet in 30 min or less for most participants [45]. To complete cpar24, using an interactive calendar the system guides study participants to select, in chronological order, specific activities carried out on the previous day (from midnight to midnight). Participants select from 262 individual activities that are arranged in 13 major categories. Once an activity is selected, the respondent is asked to specify the start and end times of the activity in durations of 5 minutes or more. Twenty-three activities allow respondents to rank their level of effort for the activity as light, moderate, or vigorous and this information is used to assign more specific MET levels for these activities. Activities that can be carried out either standing or sitting or both standing and sitting include a response option for specifying the proportion of standing and sitting times on a scale from 0 to 100%. Complete data entry is facilitated by informing the respondent about potential time gaps with the opportunity of adding missing activity items to achieve the anticipated full 1440 min/day of logged activities. Each activity reported is assigned MET value based on the 2011 Compendium [38], allowing for estimation of energy expenditure. The cpar24 can be administered several times over the course of a year to account for seasonal variation in activity participation. The tool is currently being used to assess activity and sedentary behaviors in the German National Cohort (GNC or NAKO Gesundheitsstudie), a population-based prospective study of 200,000 women and men aged 20–69 years residing in Germany that began in 2014 [46]. The cohort will be followed prospectively for ascertainment of newly incident diseases for many years. Written informed consent is obtained from all study participants and the study was approved by the relevant Ethics Committees. The present analysis is based on data from 1874 men and 1617 women from Regensburg, Germany. The cpar24 was evaluated for validity in comparison to the ActiGraph device [45].

### Time-use Categories and Energy Expenditure

The comparability of the instruments, methods and applications made it possible to examine the similarities and differences in the classification of activities and time-periods of the day (e.g. sleep/in-bed, waking day), as well as the relevant time-use allocations and estimates of energy expenditure. In terms of time use, we classified time into several categories including overall time and sedentary and active time, across personal care (bathing, dressing, grooming, toilet, eating, etc), paid work, household chores and caring activities (cooking, cleaning, caring for others, food shopping, and other non-discretionary time outside of work), transportation (automobile, bus, train, or walking and/or cycling for transportation), and leisure-time (social, relaxation, sports, exercise, etc). For energy expenditure, we calculated total energy expenditure (sleep, sedentary, physical activity), and physical activity energy expenditure (sum of light, moderate, vigorous activity). Instrument specific definitions for sedentary, light, moderate, and vigorous intensity activity are provided in Table 1.

## Results

Table 1 highlights key characteristics of the previous-day recall instruments and the study populations in which they were administered. Two of the instruments were administered using computer-assisted telephone interviews (PAR, MARCA) while ACT24 and cpar24 were self-administered using a personal computer. A variety of contextual categories were used, and each tool relied upon the Ainsworth Compendium with some variation in the version used. Typical recall completion time was 15 to 30 min as recorded by study staff or the computer-based system, and participants reported an average of 23 to 33 activities per recall. The PAR, MARCA, and cpar24 used a strict 1.5 METs threshold to differentiate between sedentary time and light intensity activity, while ACT24 applied a classification that used body posture information for lower intensity activities and classified motorized transportation as sedentary. The study participants were from the United States (Pennsylvania [PA], Iowa [IA]), Australia and New Zealand (AU), and Germany (DE).

### Participant characteristics and overall time-use

Table 2 presents additional detail about each study population and the overall amount of time reported in the major time-use categories for more than 8000 participants across all studies. The populations in which the MARCA was used were somewhat younger (early 30s), while the populations in which the PAR and cpar24 were used had a mean age of about 50 yrs., and adults who completed ACT24 were older (early 60s). The prevalence of obesity was highest in the US studies. The length of the waking day (out of bed) ranged from 15.4 to 16.1 h/d.

**Table 2:** Participant characteristics and description of overall time-use from each instrument: study

	ACT24: PA		PAR: IA		MARCA: AU		cpar24: DE	
	Men	Women	Men	Women	Men	Women	Men	Women
Sample size (N)	508	512	615	853	1018	1289	1874	1617
Participant Characteristics								
Age (yrs)	63.9 (5.8)	62.3 (6.1)	48.1 (13.1)	51.3 (11.9)	34.0 (21.2)	32.6 (19.0)	49.8 (12.4)	48.6 (12.2)
Height (cm)	176.3 (6.7)	162.7 (6.1)	177.1 (7.1)	162.8 (7.0)	177.9 (7.3)	164.8 (6.9)	178.5 (7.0)	165.2 (6.5)
Weight (kg)	88.3 (15.1)	73.4 (14.2)	94.9 (21.4)	81.6 (21.7)	78.3 (15.6)	64.5 (14.6)	86.4 (14.7)	70.3 (14.6)
BMI (kg/m2)	28.4 (4.2)	27.7 (4.9)	30.3 (6.5)	30.9 (8.2)	24.9 (4.0)	24.3 (5.4)	27.1 (4.4)	25.8 (5.3)
Obese (%)	29	32	44	45	10	14	21	19
Overall Time-use, by Category (hrs/d)								
In-bed/sleep	7.9 (1.0)	8.1 (1.1)	7.9 (1.8)	8.1 (1.8)	8.5 (2.1)	8.6 (2.0)	8.2 (1.8)	8.4 (1.5)
Out-bed/waking	16.1 (1.0)	15.9 (1.1)	16.1 (1.8)	15.9 (1.8)	15.5 (2.1)	15.4 (2.0)	15.8 (1.8)	15.6 (1.5)
Waking day								
Personal care	2.3 (0.8)	2.2 (0.8)	1.6 (0.9)	1.8 (0.9)	1.9 (1.1)	2.1 (0.9)	2.3 (1.1)	2.4 (1.0)
Paid work/school	2.2 (2.6)	2.0 (2.5)	4.3 (4.3)	3.3 (4.1)	3.7 (3.5)	3.7 (3.4)	4.0 (4.1)	2.6 (3.5)
Household chores/caring	2.9 (1.8)	3.7 (1.9)	3.9 (3.3)	5.1 (3.4)	1.6 (1.9)	2.2 (2.2)	2.1 (2.2)	3.8 (2.5)
Transportation	1.5 (0.9)	1.3 (0.8)	1.3 (1.2)	1.1 (0.9)	1.8 (1.5)	1.8 (1.4)	1.5 (1.6)	1.4 (1.4)
Leisure-time	6.9 (2.4)	6.2 (2.2)	4.9 (3.3)	4.5 (2.9)	6.2 (3.3)	5.7 (3.0)	5.7 (2.9)	5.5 (2.5)

Values are mean (SD) and percentage (%)

In most studies, participants reported about 2 h/d in personal-care activities, and time spent in paid work or school activities was lowest in the older US population (ACT24, 2.0–2.2 h/d) and higher in the other studies (2.6 to 4.3 h/d). Time spent in household chores and caring activities was 28–81% greater in women than men and was somewhat lower in the younger Australian/New Zealand population. For example, women using the MARCA tool reported a mean of 2.2 h/d of household activity while German women reported 3.8 h/d in this category via the cpar24. Time spent in transportation accounted for 1.1 to 1.8 h/d across studies. Leisure-time was often the largest block of time reported. Men reported 4–11% more leisure time than women.

### Time spent in sedentary behavior and physical activity

**Fig. 1:**
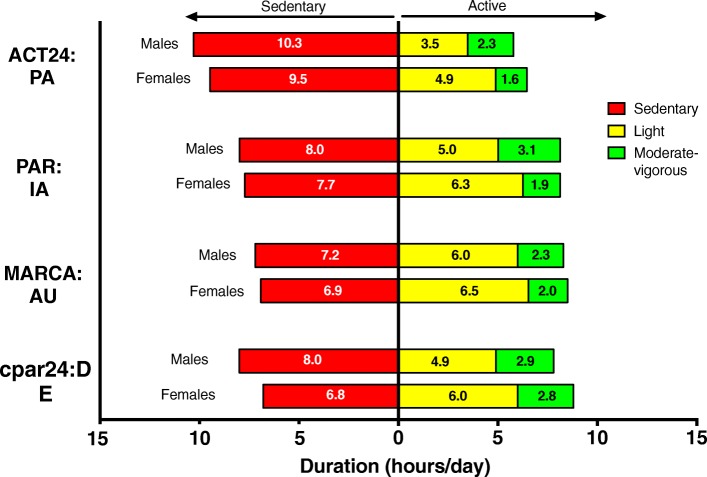
Time in sedentary, light, and moderate-vigorous physical activity, by instrument/study and sex

Figure 1 describes time spent during the waking day in sedentary behavior and physical activity, by activity intensity. The ACT24 tool captured about 10 h/d of sedentary time and 6 h/d of active time, while the other instruments captured 6.8 to 8.0 h/d of sedentary time and 7.8 to 8.8 h/d of physically active time. Examination of sex differences showed that women reported 4–15% less sedentary time, 8–40% more light intensity activity, and 3–30% less moderate-vigorous intensity activity than men. All instruments employed the 3 MET threshold to define moderate-vigorous intensity activity, and values ranged from 1.6 h/d (ACT24, women) to 3.1 h/d (PAR, men) across studies.

**Fig. 2:**
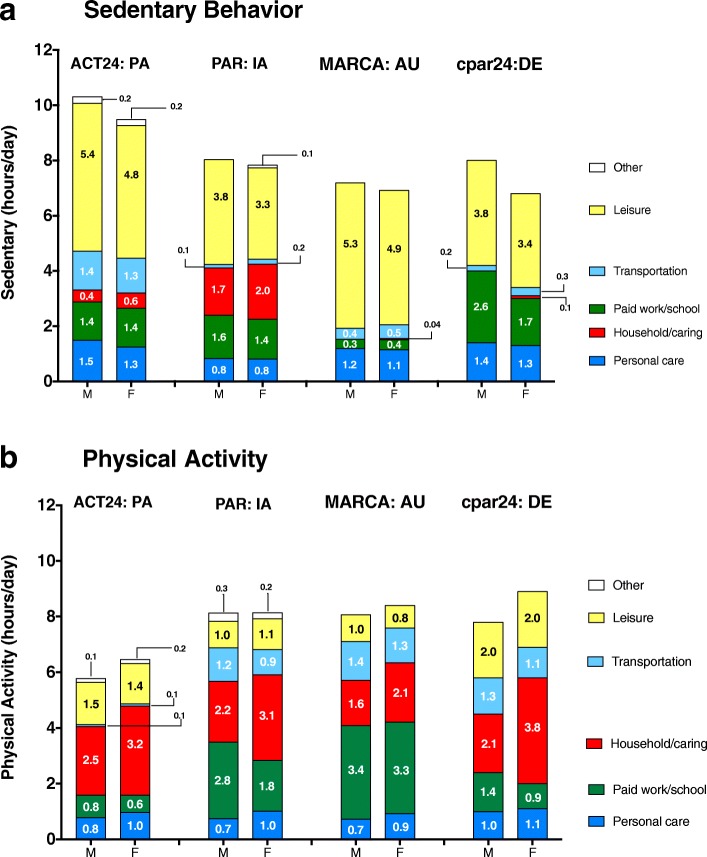
Time in sedentary behavior and physical activity by instrument/study, sex, and time-use category

Figure 2 reports time spent in sedentary behavior and physical activity, by the major time-use categories. A striking feature of sedentary behavior across all studies is that most sitting each day was reported during leisure time—often accounting for 50% or more of total daily sedentary time (Fig. 2, panel a). Women generally reported less leisure-time sedentary behavior than men. For example, in PAR, men reported 3.8 h/d in leisure sitting on the PAR while women reported 3.3 h/d. Other substantial contributors to sedentary time were paid work or school, personal care. Motorized transportation contributed 1.3 to 1.4 h/d to sedentary time in ACT24 in older US adults. This behavior was classified as a light intensity activity in the other studies and their instruments, which accounts for much of the difference between ACT24 and the other instruments in total sedentary and active time (see below).

Leisure-time physical activity was only a modest contributor to total active time (Fig. 2, panel b). In three out of four studies, women reported more total physical activity than did men, and most of this difference was due to greater amounts of household and caring activities reported by women across all studies. Paid work/school activities were also a substantial contributor to total activity. The amount of time reported in personal care (0.7 to 1.1 h/d) was relatively consistent across all studies, and time spent in transportation ranged from 0.9 to 1.4 h/d in the PAR, MARCA and cpar24 instruments.

### Total and physical activity energy expenditure

**Fig. 3:**
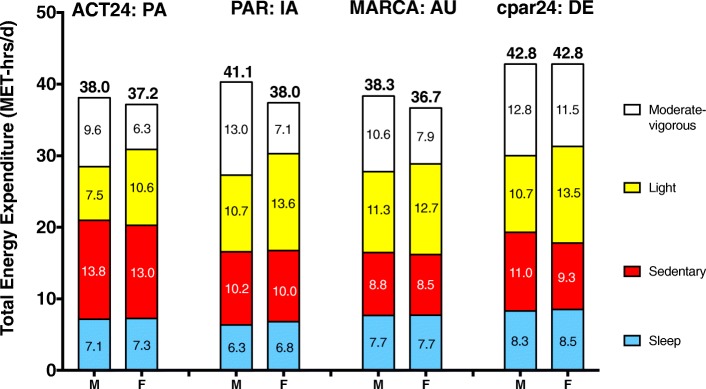
Total energy expenditure (MET-hrs/d), by instrument/study, sex, sleep, sedentary, and physical activity

Figure 3 presents estimates of total energy expenditure (MET-hrs/d), with specific estimates for sleep, sedentary, light, and moderate-vigorous intensity activity. The largest estimates of TEE were from men from Germany (cpar24, 42.8 MET-hrs/d) and Iowa (PAR, 41.1 MET-hrs/d), while TEE values from the other study and sex groups clustered around 37 MET-hrs/d (range, 36.7 to 38.3 MET-hrs/d). Men tended to expend more energy in moderate-vigorous intensity activity than women, and light intensity activities made a substantial contribution to total expenditure in all four studies. Dividing estimates of TEE (MET-hrs/d) by 24 h approximates the physical activity level (PAL) metric commonly used in doubly labeled water studies (PAL = TEE/Resting energy expenditure), and PAL estimates across the present studies ranged from 1.53 among Australian/New Zealand women (MARCA) to 1.78 for German adults (cpar24).

**Fig. 4:**
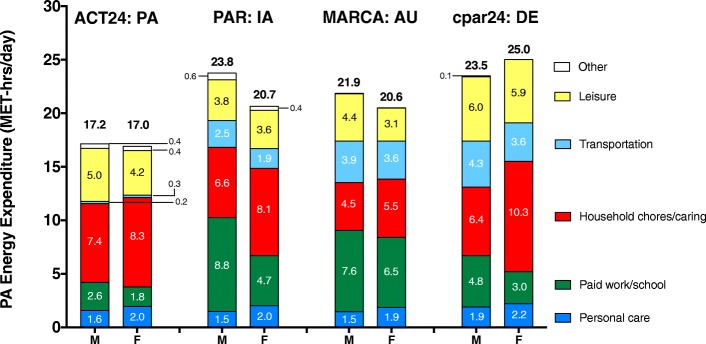
Physical activity energy expenditure (MET-hrs/d), by instrument/study, sex, and time-use category

Figure 4 presents estimates of physical activity energy expenditure (PAEE, excluding expenditure in sleep and sedentary behavior) by study and time-use category. German adults reported the most PAEE (cpar24), followed by adults from Iowa (PAR) and Australia/New Zealand (MARCA), and values were lowest for older US adults, primarily because of the classification of motorized transportation as a sedentary rather than an active behavior in the ACT24 instrument. The most prominent sources of PAEE were from Household chores/caring, Paid work/school, and leisure sources. For example, among older US adults who completed ACT24, 4.2 to 5.0 MET-hrs/d of leisure-time PAEE was reported, representing about 25 to 30% of total PAEE, a proportion comparable to that observed in the cpar24 results. PAEE in household/caring activities was greater in women than men in all four studies.

## Discussion

This paper describes four previous-day recall instruments that were designed to assess physical activity and sedentary behavior and to estimate energy expenditure using either telephone interviews or computer-based methods for self-administration at home. The instruments were administered to more than 8000 participants in studies conducted in the US, Germany and Australia and New Zealand. Better quality time-use diaries are thought to capture a large number and wide range of daily activities [47], and the methods examined here captured a large number of reported activities per recall (average, 23 to 33 activities per recall) distributed across the major time-use categories evaluated. The instruments provided broadly comparable estimates of overall time use, total energy expenditure, moderate-vigorous intensity physical activity, and consistent and expected differences by sex were noted in each study (e.g., sedentary behavior, light activity, household chores). These findings suggest much commonality between methods, even though each instrument was developed independently, and we only worked to harmonize readily available summary output from each instrument for this report. The main differences between methods revolved around operationalizing the definitions of sedentary behavior and light intensity physical activity, and thus the balance of total time in these behaviors. The remainder of this discussion considers unique aspects of previous day recalls for assessment of physical activity and sedentary behavior, the validity of the methods, key issues to address for harmonization, and needs for future research.

Time-use surveys, and the previous-day physical activity recalls evaluated in this report, are both designed to capture a profile of daily life by asking participants to report the duration of the many different episodes of activity they did yesterday, and a series of follow-up questions designed to meet the goals of the research instrument. Physical activity instruments have historically focused on maximizing the accuracy and precision of estimating energy expenditure, by using activity categories characterized by a single MET value, particularly for paid and unpaid work and leisure time activities, especially exercise/sports activities. Additionally, physical activity-oriented instruments often collect more explicit information about body posture and they are also beginning to incorporate additional information (i.e., type, purpose, location) to place behavior in context [48, 49]. The PAR instrument is a good example of this evolution (see Table 1). In contrast, time-use surveys seek to characterize the social and economic functions of time use in greater detail. For example, for the American Time-use Survey (ATUS) participants are often asked who they were with while doing an activity, where the activity took place, and much detail about the economic impacts of the activity, including for example, classification of up to 16 different reasons for travel [50].

Our previous-day recall methods may be particularly useful for estimating the use of time and energy in lower intensity activities of daily life, such as household activities and personal care. Energy expenditure estimates for women depend in significant part on household activities [52], even though time devoted to such activities has declined in recent decades [1, 51]. Household activities have long been known to be difficult to measure via questionnaire [7] but household production has historically been an important target for time-use surveys [1, 21]. Both time-use and previous-day physical activity recalls may be particularly well suited to assess these common lower intensity daily behaviors because the activities are captured with a similar level of detail in both types of instruments, and most of these activities are done while standing. All four of our studies showed household activities and caring to be substantial contributors to overall physical activity energy expenditure, and, as expected, women reported doing more of this type of activity than men [52, 53]. There is currently much interest in the physical activity community to understand the possible health benefits of lower intensity activities of everyday living, and previous-day recalls have the potential to provide important insights in future studies. Indeed, results from this study suggest that adults spend most of their physically active time in light intensity activity and that the amount of energy expended in light activity is substantial. Women reported expending more energy in light intensity activity than in moderate-vigorous activity, while men expended only a bit more energy in moderate-vigorous intensity activity than in light activity (Fig. 1, Fig. 3).

An important strength of this report is that two of the instruments have been evaluated for test-retest reliability [42, 45], and all have been validated in free-living studies against strong criterion measures. In comparison to doubly labeled water (DLW), ACT24 and MARCA provided estimates of TEE within 50 to 83 kcal/d (2–3%) of DLW [33, 44], while the PAR underestimated TEE by only 228 kcal/d (− 8%) [40] compared to a validated accelerometer [54]. Estimates of PAEE from ACT24 and MARCA were within − 105 to 75 kcal/d (− 6 to 10%) of PAEE estimates from DLW [33, 44]. As a group, the instruments examined in this report had PAL values of 1.53 to 1.78, which is entirety consistent with the PAL levels of free-living adults (18–64 yrs) in affluent societies, which range from 1.64 to 1.85 [55]. The instruments evaluated here have been found to be significantly correlated with TEE (r = 0.70 to 0.87) [33, 40, 44], PAEE (r = 0.56 to 0.63) [33, 44], sedentary time (r = 0.49 to 0.70) [33, 45, 49, 56], light intensity activity (r = 0.34 to 0.46) [45], and moderate-vigorous intensity activity (r = 0.47 to 0.59) [33, 40, 45] in high quality validation studies.

Over-reporting of physical activity is always a concern with self-report measures, however understanding if and/or how much reporting bias exists is more complex than typically appreciated. A notable result in this report was that all four instruments found relatively high levels of moderate-vigorous intensity physical activity (1.6 to 3.1 h/d across studies), values much higher than estimates from first generation accelerometer that were calibrated only to assess ambulatory activities [57, 58] that have come to define our understanding of the amount of moderate-vigorous intensity activity accumulated in daily life. There are several potential reasons for this finding, including the possibility that the estimates reported herein are reasonably accurate. First, it is generally believed that over-reporting of physical activity is common due to social desirability biases [15], yet the previous-day recall method was adopted to minimize these types of biases, and two studies that directly tested this hypothesis found no evidence of social desirability biases for previous-day recall methods [31, 59].

Second, there are two methodological issues that could also contribute to apparently higher estimates of moderate-vigorous physical activity. A cut-point bias favoring more moderate-vigorous activity could arise due to asymmetry in the MET values of reportable activities on the previous-day recalls since there tend to be more activities at or just above the 3 MET moderate intensity threshold than just below it (i.e., in the 2.3 to 2.9 MET range). Inter- and intra-individual variability will mean that some activities notionally requiring 3 METs will require less, and hence will register as device-measured moderate-vigorous activity. In addition, the minimal reporting epoch of 5 min on the recalls could also contribute to apparent over-reporting, particularly when longer duration episodes of activity (e.g., 45 min) are reported without considering short breaks that can naturally occur during an episode of activity.

Third, it is possible that the recall-based estimates of moderate-vigorous intensity activity reported here are reasonably accurate. Most of our knowledge about the amount of moderate-vigorous intensity activity accumulated by adults has come from first generation accelerometers calibrated in the laboratory on treadmills using only ambulatory activities [57, 60, 61], even though free-living indirect calorimetry studies suggest these methods may substantially underestimate moderate-vigorous activity [62, 63]. Matthews and colleagues [61] recently reported that accelerometer-based methods calibrated to both lifestyle and ambulatory activities may capture as much as 90% more moderate-vigorous activity (1.82–2.28 h/d) compared to methods calibrated to ambulatory activities alone (0.35–0.97 h/d). In this study, ACT24 estimates of moderate-vigorous activity were similar to (men) or lower than (women) the more broadly calibrated accelerometer methods, and detailed data from ACT24 show that participants reported engaging in a broad-range of moderate intensity lifestyle activities. Similarly, in PAMS, participants reported spending approximately 2.4 h/d in moderate-vigorous intensity activity and this was similar to the amount recorded by the SenseWear Armband (2.2 h/d), a multi-sensor device with documented evidence of validity for assessing lifestyle activity and total energy expenditure [54]. More studies are needed, but these observations are consistent with the idea that adults may participate in as much moderate-vigorous intensity activity as they say they do on previous-day recalls. Future validation studies of time-use measures evaluating moderate-vigorous intensity activity are encouraged to use criterion measures designed to capture the full-range of daily activities (e.g., [64, 65]).

While the four instruments evaluated were relatively consistent in capturing total energy expenditure, moderate-vigorous intensity physical activity, and household activities, substantive differences were noted in estimates of time spent in sedentary behavior and light intensity physical activity. The PAR, MARCA, and cpar24 estimated participants spent 7 to 8 h/d in sedentary behavior and 8 to 9 h/d in physical activity, while ACT24 estimated participants spent about 10 h/d sedentary and 6 to 7 h/d in physical activity. Although some of this difference could be due to the older age of participants in the iDATA study (ACT24), we believe that most of this effect resulted from how definitions of sedentary behavior and light intensity activity were operationalized when applying scoring algorithms to the 200 to 500 different activities reportable across studies. Although the definition of sedentary behavior proposed by the Sedentary Behavior Research Network appears straightforward (i.e., any waking behavior characterized by an energy expenditure ≤1.5 METs, while in a sitting, reclining or lying posture, [37]), there was some variation in how it was applied between studies. Three instruments (PAR, MARCA, cpar24) that captured less sedentary time and more activity focused on the 1.5 MET threshold portion of the definition to make this classification, while the instrument that captured more sedentary time and less activity (ACT24) placed more emphasis on body posture for lower intensity activities, and classified riding in or driving a vehicle as sedentary even though MET levels for these activities are 2.0 METs or greater in the Ainsworth Compendium. These substantive differences are ripe for consensus work that could reduce such differences in future studies to take greater advantage of the rich contextual detail provided by previous-day recalls (see next section).

## Conclusions

The four previous-day recall instruments examined in this report were found to provide comparable estimates of total energy expenditure, moderate-vigorous intensity physical activity, and patterns of time use in relevant categories (i.e., housework, leisure-time activity) and each has been validated in free-living studies. The major differences noted between instruments were in how the definitions of sedentary behavior and light intensity physical activity were operationalized for each instrument, resulting in relatively large differences between studies in sedentary and active time, as well as the allocation of time in specific time-use categories. Improving comparability has long been a goal of time use surveys [66] and several steps could be taken to do so with respect to physical activity measurement. High priorities in this area include efforts to improve behavioral classification (e.g. sedentary behavior vs light activity) and better asses intensity (e.g. light versus moderate) in free-living populations. The first step would be to establish a core list of activities embedded in the recall system that could be selected by participants and/or interviewers during completion of the recall, preferably with a consistent approach to identifying body posture to aid in classifying sedentary and active behaviors. Second, would be establishing a common approach to linking reportable activities and their posture to MET values in the Compendium. This task is relatively straightforward for activities that can be matched on a 1:1 basis (e.g., walking or running for exercise), but it is complicated when the selectable activity is a composite of several related but different activities (e.g., “food preparation and serving” may include chopping, cooking, washing dishes, setting the table and serving food). For composite activities, there are often several logical linkage choices in the Compendium, and variation in these choices can result in major classification differences (e.g., sedentary, light, moderate activity). While the Compendium is an extraordinary resource that has done more to standardize the assessment of physical activity than any other single tool, assigning MET values consistently to lower intensity activities that could involve sitting or standing postures may stretch the limits of precision for activities with MET values in the range of 1 to 2 METs, given its reliance on data sources that did not always quantify the effect of body posture on the energy cost of individual activities. Third, once the core information is assembled (activities, posture, METs), the next step would be to determine a consistent approach to translate this information in the relevant behavioral classifications (sleep, sedentary, active) and domains of living, or time-use categories (e.g., work, travel, leisure). Finally, information about activities could be extended by capturing relevant attributes about each behavior as they are reported, including details about the location, social context, purpose, or response to the activity (e.g., mood, indicators of well-being).

Previous day recall instruments designed to assess physical activity behavior provide considerable value for a number of different research and surveillance applications. The ability of these tools to capture the type of activity provides valuable context to both understand and influence behaviour. People construe their day in terms of activity domains (e.g. chores, TV) rather than as energy expenditure bands (light PA, MVPA), so this information enables more specific and individualised recommendations for time re-allocation. Furthermore, these tools provide information that aid in both intervention design and evaluation (e.g. who an activity is done with, where it is done and potentially how much it is enjoyed). The comparison of these four different instruments in the present study highlight ways to standardize and harmonize outcomes from these tools. Progress is also needed in improving methods to estimate energy expenditure from existing and future time-use surveys and regression-based calibration methods that adjust and re-scale reported estimates of physical activity has documented potential in this regard [39]. Lastly, ongoing work is required to adapt and update these instruments to changes in technology. Considerable work has been invested in refining and calibrating accelerometer-based methods over the years and this has led to systematic advances in the utility of these methods. Parallel efforts to optimize and further improve previous-day recall methodologies has the potential to provide similar dividends.

## Abbreviations

ACT24: Activities Completed over Time in 24 h
CATI: Computer assisted telephone interview
cpar24: Computer-based 24-h Physical Activity Recall
DLW: doubly labeled water
iDATA: Interactive Diet and Activity Tracking in AARP
MARCA: Multimedia Activity Recall for Children and Adults
PAEE: physical activity energy expenditure
PAMS: Physical Activity Measurement Survey
PAR: Physical Activity Recall
TEE: total energy expenditure
